# Allergic Bronchopulmonary Aspergillosis: A Case Report

**DOI:** 10.7759/cureus.38778

**Published:** 2023-05-09

**Authors:** Siham Bouali, Imane Legssyer, Afaf Thouil, Hatim Kouismi

**Affiliations:** 1 Department of Respiratory Diseases, Mohammed VI University Hospital, Faculty of Medicine and Pharmacy, Mohammed First University, Oujda, MAR; 2 Department of Respiratory Diseases, Mohammed VI University Hospital, Oujda, MAR; 3 Research and Medical Sciences Laboratory (LRSM), Faculty of Medicine and Pharmacy, Mohammed First University, Oujda, MAR

**Keywords:** antifungal therapy, oral corticosteroid, immunoglobulin e, bronchiectasis, asthma, allergic bronchopulmonary aspergillosis

## Abstract

Allergic bronchopulmonary aspergillosis (ABPA) is an underdiagnosed lung condition in patients with asthma and cystic fibrosis. Its clinical and diagnostic manifestations result from an allergic response to multiple antigens expressed by *Aspergillus fumigatus*, which colonize the bronchial mucus. This report presents the case of a 73-year-old female patient referred to our hospital for uncontrolled asthma for 35 years. The diagnosis of ABPA was made on the basis of clinical symptoms, peripheral blood eosinophilia, elevated total serum immunoglobulin E, positive aspergillus serology, and bronchiectasis with mucoid impaction. Systemic corticosteroids and antifungal therapy came up with satisfactory clinical results.

## Introduction

Allergic bronchopulmonary aspergillosis (ABPA) is a rare inflammatory lung disease, characterized by an abnormal reaction of the immune system against Aspergillus that colonizes the airways. This disease mainly affects asthma patients or those suffering from cystic fibrosis and can exceptionally affect healthy people [1]. Indeed, these diseases are associated with a production of viscous mucus and an impairment of its elimination, allowing inhaled Aspergillus particles to persist in the airways.

It remains underdiagnosed in many countries, especially in the developing world, and may be misdiagnosed as pulmonary tuberculosis in up to one-third of cases [2]. The delay in diagnosis is long and can be up to 10 years between the appearance of the first symptom and the identification of ABPA [3].

We report the case of a patient followed for refractory asthma in whom, after several investigations, the diagnosis of ABPA was made.

## Case presentation

The patient was 73 years old and had been treated for uncontrolled asthma on long-acting beta2-agonists (LABAs)+Inhaled corticosteroids (ICS) for 35 years, with frequent hospital admissions. She had no history of tuberculosis and was admitted to the emergency room with resting dyspnea and a cough that produced mucopurulent sputum. These symptoms were associated with an intermittent fever and a deterioration of general condition.

On clinical examination, the patient was conscious, with a normal blood pressure of 110/70 mmHg, tachycardia at 100 beats per minute, oxygen saturation of 60% on room air becoming 92% under 7L of oxygen, and apyretic at 37.2°C. On auscultation, she had bilateral rhonchi.

Blood tests revealed a white blood cell count of 18,000/mm3 with a neutrophil predominance, a c-reactive protein (CRP) of 200 mg/l, and a d-dimer level of 1500 ng/ml.

**Figure 1:**
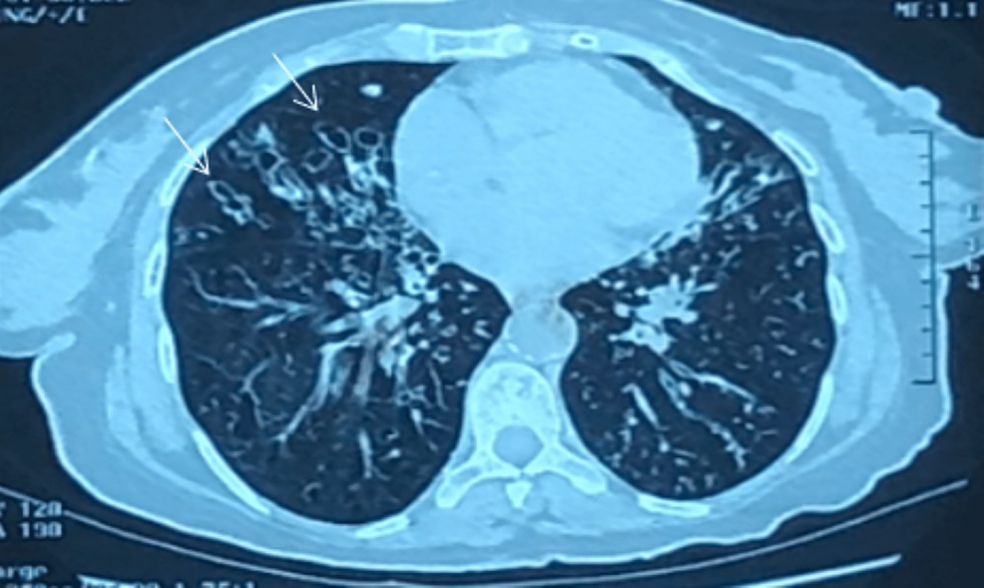
Chest CT scan showing bilateral cystic and cylindrical bronchial dilatation (white arrows) with mucoid impaction

The CT angiography of the chest ruled out pulmonary embolism and showed a cystic and cylindrical bilateral bronchiectasis site with mucoid impaction (Figure 1).

Arterial blood gas test showed a respiratory acidosis with a pH of 7.24 and a partial pressure of carbon dioxide (PaCO_2_) of 93 mmHg.

During her hospitalization, the patient was put on non-invasive ventilation with an initial FIO_2_ of 40% and on antibiotic therapy based on third-generation cephalosporin. The evolution was marked by a clinical, biological, and gasometric improvement with a control pH of 7.37 and a PCO_2_ of 55 mmHg.

Once the patient was stabilized on the respiratory level, an etiological investigation in the context of her bronchial dilatation was carried out with an acid-fast bacilli sputum smear and cultures, an immunoglobulins blood test, serum total immunoglobulin E (IgE), Aspergillus fumigatus-specific IgE, and Aspergillus serology. This revealed an elevated serum IgE level of 550 IU/mL, hypereosinophilia of 600 cells/μL, and a positive aspergillosis serology (1/640).

Based on the serum level of IgE, aspergillosis serology, hypereosinophilia, history of poorly controlled asthma, and the presence of bronchiectasis on chest CT, a diagnosis of allergic bronchopulmonary aspergillosis was made. The patient was put on oral corticosteroids based on prednisolone (1 mg/kg per day for two weeks and then a reduction of 10 mg every two weeks for five months) and itraconazole (a loading dose of 800 mg the first day and then 200 mg twice a day) after normal liver function tests. The patient also received nebulization sessions with salbutamol.

She was discharged from the hospital after 20 days with clinical improvement of her respiratory symptoms.

## Discussion

ABPA was first described by Hinson and colleagues in 1952 and is often associated with chronic lower airway diseases (asthma and cystic fibrosis) which are associated with viscous mucus production and impaired mucociliary clearance, allowing inhaled Aspergillus fumigatus spores to persist in the airways. It may occur infrequently in patients without a history of bronchial asthma [4].

A study concluded that 2.5% of adults with asthma also have ABPA, which is about 4.8 million people worldwide [5].

There is no age or gender predilection for its development [5]. Sputum with brown mucus plugs is a suggestive symptom, but it is seen in only 31 to 69% of patients [6]. On auscultation, the most common finding is wheezing [6]. Physical examination may also reveal signs of pulmonary hypertension and respiratory failure.

The diagnosis of ABPA is based on immunological investigation. An elevated level of Aspergillus fumigatus-specific IgE with a level above 0.35 kUA/l is currently the most sensitive test for the diagnosis of ABPA and is also considered the preferred test for screening of ABPA in asthmatic patients [7]. With regard to the Aspergillus skin test, it is not currently preferred for screening ABPA in patients with asthma. It has a sensitivity of 88% to 94% and therefore can potentially miss 6% to 12% of patients with ABPA [7,8]. Measurement of serum total IgE can be an effective diagnostic tool for patients with ABPA, and it is also useful for subsequent follow-up. If a patient's serum total IgE level is normal, this largely rules out active ABPA as the underlying cause of their symptoms. However, serum total IgE (with a cutoff of 500 IU/mL) has a high sensitivity (96%) but low specificity (24%) for identifying ABPA in asthmatic patients [8]. Therefore, it is not a recommended screening test for ABPA.

Regarding radiological investigations, high-resolution chest CT is currently the preferred imaging modality for ABPA [9]. The central bronchiectasis are characteristic and of frequent discovery but they can reach the periphery in about 40 % of the cases. ABPA can occur without any radiological manifestation, thus, It can be diagnosed on an immunological basis only. The importance of MRI in the diagnosis of ABPA is still being evaluated and, therefore, it is not recommended in routine practice [10].

The lack of an agreed-upon standard has led to the suggestion of numerous criteria over time. Of these, the Rosenberg-Patterson criteria have gained the greatest acceptance in asthma-related ABPA diagnosis [11,12]. However, a new set of criteria was proposed by the ISHAM-ABPA working group and published in 2013, as documented in Table 1 [13].

**Table 1:** New proposed diagnostic criteria for ABPA from the 2013 ISHAM working group *If the patient meets all other criteria, an IgE value of less than 1000 IU/mL may be acceptable. *Radiological features consistent with ABPA may be temporary (infiltrates, nodules, glove finger opacities) or permanent (parallel lines in rails or cocoons, bronchial dilatation and pleuropulmonary fibrosis). ABPA: Allergic bronchopulmonary aspergillosis

Predisposing factors	Asthma, cystic fibrosis
Obligatory criteria	-Positive skin test for Aspergillus fumigatus (type I hypersensitivity reaction, immediate hypersensitivity) or elevated specific IgE antibodies to Aspergillus fumigatus.
	-Elevated total IgE level above 1000 IU/mL*.
At least two of the following three criteria	- Presence of precipitating or IgG antibodies against Aspergillus fumigatus in serum.
	-Lung opacity suggestive of ABPA*.
	-Hypereosinophilia above 500/mm3 in patients who have not received corticosteroids.

Recently, the Japan ABPM research program introduced ten new diagnostic criteria for ABPM/ABPA in patients without cystic fibrosis (Table 2). A diagnosis of ABPA requires meeting at least six of the specified criteria. According to the study, the new criteria claim better sensitivity and specificity for detecting ABPA than the previous criteria [14].

**Table 2:** Asano criteria for ABPA ABPA: Allergic bronchopulmonary aspergillosis

1-Current or previous history of asthma or asthmatic symptoms
2-Peripheral blood eosinophilia (≥500 cells/mm^3^)
3-Elevated total serum IgE levels (≥417 IU/mL)
4-Immediate cutaneous hypersensitivity or specific IgE for filamentous fungi
5-Presence of precipitins or specific IgG for filamentous fungi
6-Filamentous fungal growth in sputum cultures or bronchial lavage fluid
7-Presence of fungal hyphae in bronchial mucus plugs
8-Central bronchiectasis on CT
9-Presence of mucus plugs in central bronchi, based on CT/bronchoscopy or mucus plug expectoration history
10-High attenuation mucus in the bronchi on CT

Treatment of ABPA is aimed at managing the symptoms of asthma and cystic fibrosis, reducing lung inflammation, preventing or treating ABPA-related lung exacerbations, and slowing progression to fibrotic or cavitary disease that may reach a terminal stage. Therefore, early and exhaustive treatment is essential [1].

After treatment, many patients in the acute and exacerbation stages may experience complete recovery, represented by a 35% to 50% reduction in total serum IgE after six weeks, resolution of lung infiltrates, and improvement in symptoms. Unfortunately, this remission is not always permanent, as some patients relapse [15].

Corticosteroids remain the cornerstone in the treatment of ABPA and target the inflammatory response triggered by A. fumigatus [16]. The dose of oral steroids for ABPA has not been well defined. A lower dose is associated with a higher recurrence rate [16].

Inhaled corticosteroids are a central component in the treatment of persistent asthma, improving symptoms but not completely controlling them [17]. Studies concluded that high doses of inhaled corticosteroids should not be used as first-line therapy as they have no impact on the management of ABPA [18].

Antifungal therapies play an important but complementary role. By decreasing fungal colonization and mitigating inflammatory responses, they may reduce the need for prolonged high doses of systemic corticosteroids. [16].

The long-term prognosis of patients with ABPA is still unclear. Untreated patients progress to irreversible pulmonary fibrosis and respiratory failure [2].

## Conclusions

Due to the lack of standardized diagnostic and screening criteria, ABPA is still underdiagnosed and treatment is often delayed. When clinical, radiological and biological criteria are met and the diagnosis is made, treatment with both corticosteroids and an antifungal drug, itraconazole, should be initiated to avoid possible progression to respiratory failure.
